# In-Situ Preparation of Aramid-Multiwalled CNT Nano-Composites: Morphology, Thermal Mechanical and Electric Properties

**DOI:** 10.3390/nano8050309

**Published:** 2018-05-07

**Authors:** Jessy Shiju, Fakhreia Al-Sagheer, Ali Bumajdad, Zahoor Ahmad

**Affiliations:** Chemistry Department, Kuwait University, PB. 5969, Safat-13060, Kuwait; jessyshiju82@gmail.com (J.S.); f.alsagheer@ku.edu.kw (F.A.-S.); bumajdad@gmail.com (A.B.)

**Keywords:** polyamides, CNTs, nano-composites, interfacial bonding, visco-elastic and electric behavior

## Abstract

In this work in-situ polymerization technique has been used to chemically link the functionalized multiwalled carbon nanotubes (CNTs) with aramid matrix chains. Phenylene diamine monomers were reacted in the first stage with the carboxylic acid functionalized CNTs and then amidized in-situ using terephthaloyl chloride generating chemically bonded CNTs with the matrix. Various proportions of the CNTs were used to prepare the hybrid materials. The functionalization procedure was studied by Fourier transform infrared (FTIR) spectroscopy and composite morphology investigated by scanning electron microscopy (SEM). Thermal mechanical properties of these hybrids, together with those where pristine CNTs with similar loadings were used, are compared using tensile and dynamic mechanical analysis (DMA). The tensile strength and temperature involving α-relaxations on CNT loading increased with CNT loading in both systems, but much higher values, i.e., 267 MPa and 353 °C, respectively, were obtained in the chemically bonded system, which are related to the nature of the interface developed as observed in SE micrographs. The water absorption capacity of the films was significantly reduced from 6.2 to 1.45% in the presence pristine CNTs. The inclusion of pristine CNTs increased the electric conductivity of the aramid films with a minimum threshold value at the loading of 3.5 wt % of CNTs. Such mechanically strong and thermally stable aramid and easily processable composites can be suitable for various applications including high performance films, electromagnetic shielding and radar absorption.

## 1. Introduction

Carbon nanotubes (CNTs), with their unique structure and higher aspect ratio, provide an outstanding combination of higher mechanical, thermal and electric properties [1,2,3,4,5] with an additional advantage of their low density. Contrary to other fiber-reinforced composites, the frictional forces at the interface of polymer/CNT are large and, in the case of de-bonding, a significant load transfer from the matrix to the CNT can therefore be achieved [6,7]. These, therefore, are considered as ideal candidates to reinforce the polymeric matrices [8,9,10]. CNTs, however, have a high tendency towards aggregation due to their inter-tube van der Waals interactions [11]. For significant property improvement, a uniform dispersion of CNTs in the matrix is necessary. An effective dispersion of CNTs requires their de-aggregation and stabilization within the matrix [12], for which two main techniques have been used to disperse them in the polymer matrix. To break the agglomerates, mechanical stress has been employed using techniques such as ultra-sonication, calendaring, ball milling or shear mixing [13,14,15,16,17]. These methods, however, are successful only at a low concentration of CNTs, as at high concentrations, the increase in resin viscosity can make their dispersion difficult [18]. Surface treatments of CNTs using both physical and chemical methods have also been tried with the purpose of increasing the interfacial interaction with the matrix [19,20]. Functionalization of CNTs can be achieved by different processes such as fluorination, cyclo-addition, and their further reactions [19,20,21,22,23,24,25,26]. Oxidation of CNTs [27] can be achieved by chemical methods using strong mineral acids such as HNO_3_ and H_2_SO_4_. Milder approaches such as oxygen plasma, photo-oxidation, or gas phase treatments have also been tried. Chemical treatments enhance the solubility of CNT by providing primary or secondary bonding with the polymer chains. In recent years, aliphatic polyamides [28,29,30,31] and various other commodity polymers, e.g., epoxies [32,33,34,35] polycarbonates [36,37], polymethylmethacrylate [38] and polystyrene [39] have been used as matrices employing different techniques to prepare composites with CNTs for industrial applications. Thermal mechanical properties were found to improve in few cases [34,35,38], whereas in many reported cases [32,33,36,37,39] the value of glass transition temperature (T_g_), decreased on CNT loading, despite the fact that functionalized CNTs have been used to develop interactions with a matrix. There is therefore a need to explore the proper experimental conditions employed which can affect the nature of the resulting interphase and hence the properties of such composites.

The demand for high performance polymer composites as structural elements in aerospace light weight structures has increased in the recent years, and this has led many researchers to explore the potential applications of thermally stable polymeric composites. Polyimides [40] and aromatic polyamides (Ar) [41], owing to their mechanical strength and superior thermal stability, are increasingly being used as a matrix to prepare the nano-composites for advanced applications [42]. However, few reports are available on such advanced composites with carbon nano-fillers that can be useful for various applications such as coatings required for shielding electromagnetic interference [43], electrostatic discharge packaging [44] or radar absorption [45]. Connor et al. [46] have shown that if Kevlar fibers are soaked in a suspension of CNTs in the solvent *N*-methylpyrrolidone, this results in swelling of the fibers, thus allowing the nanotubes to diffuse into the interior of the fiber. The composites prepared using these fibers were found to be stronger and tougher than those from the original Kevlar fibers. Sainsbury et al. [47] have carried out the covalent modification of multiwalled carbon nanotubes (MWCNTs) with one and a half repeat units of the poly-p-phenyleneterephthalamide (PPTA) oligomers. However, the linear PPTA chain from which the Kevlar fiber is derived is not soluble in any organic solvent, and for this reason only oligomers [47] or the fibers [46] have been used with CNTs in previous work.

In the present work, we have modified the linear para-phenyleneterephthalamide chain from which the Kevlar fiber is derived by inserting an optimum number of kinks; meta-linkages to make it soluble in the organic solvent. A mixture of para- and meta-phenylenediamines in a molar ratio 35:65, respectively, was reacted with the functionalized CNT and terephthaloyl chloride and an in-situ polymerization technique was used to chemically bind the-functionalized carbon nanotubes with high molecular weight aramid copolymer chains used as a matrix. The functional groups of the CNTs were kept in strict stoichiometry to combine them with the monomers used for aramids. The composite films using different loadings of functionalized and pristine multiwalled CNTs were prepared in the present work. Thermal mechanical properties, water absorption and conductivity behaviors of these nano-composites films were studied. These properties are related to the nature of the interface present between the filler and the matrix due to different types of interfacial interactions, both physical and chemical. We have also discussed the discrepancies found in the experimental work in the recently reported literature where a lowering of the thermal mechanical behavior, in particular for the T_g_, has been reported regarding the inclusion of functionalized CNTs.

## 2. Materials and Methods

### 2.1. Materials

The three monomers—terephthaloyl chloride (TPC), para-phenylenediamine and meta-phenylenediamines—used in the present work were of analytical grades with a purity of 99% or more and were provided by Sigma Aldrich (Missouri, MO, USA). Anhydrous dimethylacetamide (DMAC) of 99.8% purity used as solvent in the polymerization reaction was also obtained from Sigma Aldrich. The multi-walled CNTs supplied by Nanoamor (Housten, TX, USA) had a purity equal to or more than 95%. The length of the CNTs varied from 10–30 μm. The inner diameter of these CNTs was 2–5 nm whereas the outer diameter was in the range of 10 to 20 nm. All other chemicals and reagents used in the work were of analytical grades and were obtained from Sigma Aldrich.

### 2.2. Preparation of Physically Linked AR-CNT Composite Films

A mixture of para- and meta-phenylenediamines (0.025 mol) taken in a molar ratio 35:65, respectively, was dissolved in 100 g of the solvent DMAC in a 250 mL conical flask. Ensuring completely anhydrous conditions, 0.025 mol of terephthaloyl chloride was added to react with the diamines. The polymerization reaction was allowed to complete with constant stirring of the mixture for 24 h. This resin was used as stock solution to prepare the composites with different loadings of nanotubes. Pristine CNTs (0.50 g) taken in 50 g DMAC were subjected to 3 h sonification with a further stirring for 24 h to de-aggregate the CNTs in the solvent. A measured amount of this solution was mixed in the polymer solution and stirred continuously for 24 h. The hybrid films with different CNTs loadings were cast in a Petri dish by the solvent elution at 70 °C. The films thus prepared were washed with distilled water to remove any HCl produced as by-product of the polymerization reaction. These were dried at 60 °C for 72 h and then under vacuum for 12 h at 100 °C. Aramid films with different multiwalled CNTs loadings (ranging from 0.5 to 7.5 wt %) were prepared and in this paper are being referred to as Ar-CNT-P.

### 2.3. Preparation of Chemically Bonded AR-CNT Composite Films

**Scheme 1:**
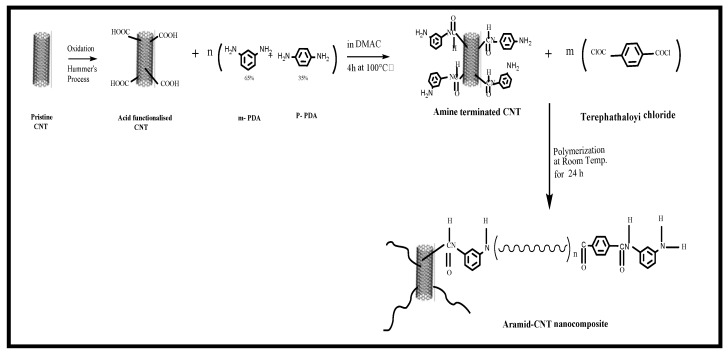
In-situ preparation of Ar-carbon nanotube (CNT)-C composites.

The pristine MWCNTs are oxidized by using nitric acid reflux at 100 °C for 4 h [27,48] and the resulting solid is washed repeatedly up to neutral pH and then dried over vacuum at 60 °C for 24 h. The presence of carboxylic functional groups was detected by FTIR. Concentration of the carboxylic groups present on the surface as a result of oxidation determined by titration against NaOH [49] was found to be 3.86 mmol/g. The required amount of functionalized CNTs was taken in a conical flask with 50 g DMAC and sonicated for 3 h. To this a mixture of para and meta-phenylenediamines, (n moles) dissolved in DMAC in mole ratio of 35:65, respectively, were added and stirred for 30 min for complete mixing. The mixture was allowed to react for 4 h at 100 °C and then cooled to room temperature. A stoichiometric amount of the monomer TPC (m moles) was added to react with the diamines to produce aramid matrix chains in the polymerization reaction (Scheme 1). The number of mols TPC were kept stoichiometric against the diamines by taking in allowance of the carboxylic groups on the CNTs reacted with diamine mixture. Composite films using different proportions ranging from 0.5 to 7.5 wt % of MWCNTs were prepared by the solvent elution technique as described in Section 2.2 and are labelled as Ar-CNT-C. Properties of these films were analyzed and these results compared with the Ar-CNT-P films where pristine CNTs were used (Section 2.2) to develop the composites.

### 2.4. Characterization of Ar-CNT Composite Films

The functional groups present on CNTs, the degree of CNT dispersion and the morphological, thermal mechanical, and tensile properties, water absorption and conductivity behavior of the composite films were studied using different techniques.

#### 2.4.1. FTIR Spectroscopy

Absorption spectra for the pristine and carboxy-functionalized CNTs powder on KBr disk were taken in the range of 400–4000 cm^−1^ using FTIR Spectrometer JASCO-6300 (Massachusetts, MA, USA) with a resolution of 4 cm^−1^ to confirm the structural differences in the two types of CNTs samples.

#### 2.4.2. UV-Visible NIR Spectroscopy

UV-Visible NIR spectra of the dilute solutions of pristine, and –COOH functionalized CNTs were recorded on Agilent 5000 UV-Vis-NIR (Santa Clara, CA, USA). The solutions were made by dissolving CNTs in DMAC with 3 h ultra-sonification followed by 24 h stirring. A few drops of this solutions taken in quartz cell were then further diluted with DMAC to obtain a light-colored solution. DMAC was used as a baseline reference and the absorption spectra were background subtracted to account for DMAC.

#### 2.4.3. Scanning Electron Microscopy

SEM was used to study the morphology of aramid composites with CNTs. The films were fractured using the microtome at −20 °C and coated with platinum. These were examined at 15 kV using SEM user Interface Model SUPRA-50 VP (JDISS) (Oberkochen, Germany). The state of the primary agglomerates of CNTs before incorporating in the matrix was also observed by SEM. The pristine multiwalled CNTs were mixed in the solvent DMAc and sonicated for 3 h and then filtered and dried and analyzed by the SEM.

#### 2.4.4. Atomic Force Microscopy (AFM)

The surface morphology of the composite films was examined by atomic force microscopy. The AFM images of the samples were obtained with a nano-scope IV scanning probe microscope (Santa Barbara, CA, USA) using contact AFM in air in the constant force mode (1 ± 2 nN). The images were acquired using the tapping mode “RTESP” tips. The cantilever was a gold coated silicon nitride with a silicon tip of 20 nm radius. The back ground was subtracted from the images using nano-scope software. A scan area of 3 μm was measured.

#### 2.4.5. Dynamic Mechanical Analysis (DMA)

The viscoelastic properties were measured on Dynamic Mechanical Analyzer Q-800 from TA (New Castle, DE, USA). The measurements were taken in the temperature range 50–500 °C in nitrogen with a floating pressure of 60 Pa kept under tension mode using a frequency of 5 Hz at a heating rate 5 min/°C. The glass transition temperature involving α-relaxations (T_g_) was measured from the peak of variation of tan δ with temperature plots.

#### 2.4.6. Tensile Analysis

Tensile analysis was performed on pure aramid and both types of composite films at 25 °C using Shimadzu Autograph TRAPEZIUM X (Kyoto, Japan) at a strain rate of 5 mm/min according to ASTM D-882 (standards).

#### 2.4.7. Electrical Properties

Electrical conductivities of the films with different CNT loadings were measured by the four-probe measuring technique under ISO 3915.1981. A standard dc current was passed between the electrodes attached by the silver paste to reduce the contact resistance. The voltage drop across the electrodes set on the films was measured using a Keithley electrometer model 2611 (Meriden, CT, USA). An average value of three measurements was taken for every film.

#### 2.4.8. Water Absorption Measurements

The amount of water absorbed in the pure aramid and the composite films was measured under ASTM D-570 (standards). Two-inch diameter films were dried in a vacuum oven for 24 h at 110 °C and then placed in a desiccator to cool and then immediately weighed. The films were merged in water at 25 °C for 48 h. The films were patted dry with a lint-free cloth and weighed and the percent of water absorption calculated.

#### 2.4.9. Thermogravimetric Analysis (TGA)

Shimadzu TGA-60H (Kyoto, Japan) analyzer was used to measure the thermal stability of composites. TGA was carried out from room temperature to 800 °C. The heating rate was kept at 10 °C/min under inert atmosphere using pure nitrogen. The first differential of the thermograms (DTGA) curves were used to measure the temperature of maximum decomposition.

## 3. Results and Discussion

### 3.1. FTIR Analysis

**Figure 1:**
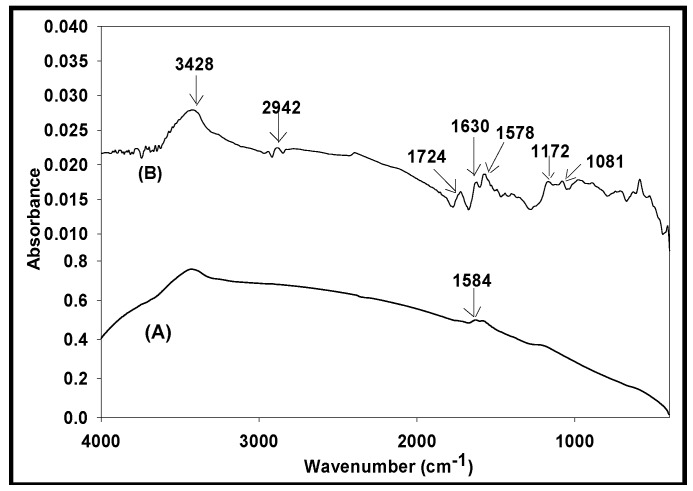
FTIR spectra: pristine (**A**) in comparison with-COOH functionalized (**B**) CNTs.

FTIR spectra of the pristine and acid functionalized CNTs powders are given in Figure 1A, and B, respectively. The presence of the peak at 1584 cm^−1^ for (C=C) bonds depicts the integrity of the hexagonal structure present in pristine CNT. The acid-functionalized MWCNT exhibits few new peaks for the polar bonds required for the physical or chemical bonding with a polar matrix. The presence of hydroxyl groups is consistent with the broad peak observed in the range of 3428 cm^−1^ and the presence of carboxyl groups at 1724 cm^−1^ [50]. The slight shift of the carboxyl peak from 1750 cm^−1^ to a lower wavelength of 1724 cm^−1^ is due to the conjugation effect by its attachment to the ring structure of CNTs wall or at tube ends [51]. The carboxyl peak also overlapped with another peak of 1630 cm^−1^ of hydrogen-bonded carbonyl stretching. This hydrogen-bonded carbonyl stretching was also spotted at 1578 cm^−1^ [52]. Besides this, the weak peaks at 1172 cm^−1^ and at 1081 cm^−1^ also appeared for acid-functionalized MWCNTs due to the C=O stretching deformation mode of the carboxylic acid group [52,53]. Another small peak at 2942 cm^−1^ is due to the (C–H) asymmetric and symmetric stretching vibration derived from long alkyl chain [50].

### 3.2. UV-Visible NIR Analysis

**Figure 2:**
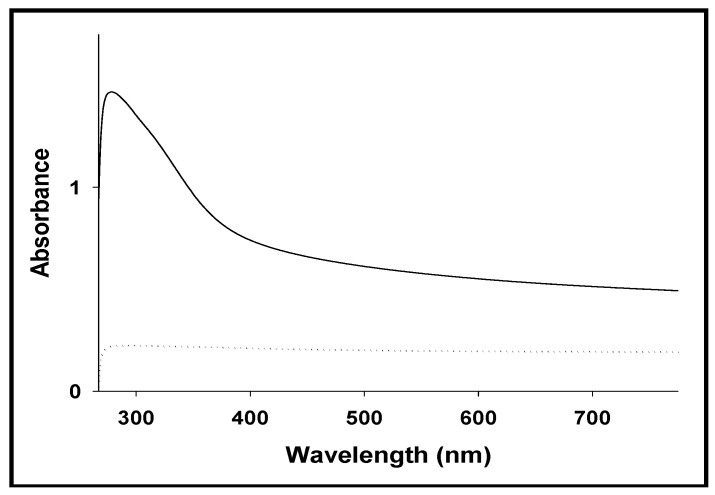
UV-visible spectra: pristine (⋯), –COOH functionalized (−) CNTs.

Individualized CNTs become active in the UV-visible region and exhibit characteristic bands due to 1D Van Hove singularities [54], whereas the bundled CNTs are not active in the wavelength region between 200–700 nm because of the tunneling effect within the tubes [55]. Spectra for UV-visible NIR absorption obtained from the dilute solution of functionalized-CNT, when compared with pristine CNTs, therefore, can give valuable information about the degree of dispersion of CNTs. The functionalized CNTs gave a strong optical absorption peak around 260–350 nm as depicted in Figure 2, whereas the pristine CNTs did not show any optical absorption in the measured UV-visible range. The increase in the absorption peak intensity indicates that the dispersion of such nanotubes can be enhanced in comparison to the pristine one in the solvent due to the presence of functional groups on the CNT surface: this is in good agreement with other evidence discussed further in this paper which proves the effect of modification on the dispersion of CNTs in the matrix.

### 3.3. Scanning Electron Microscopy

**Figure 3:**
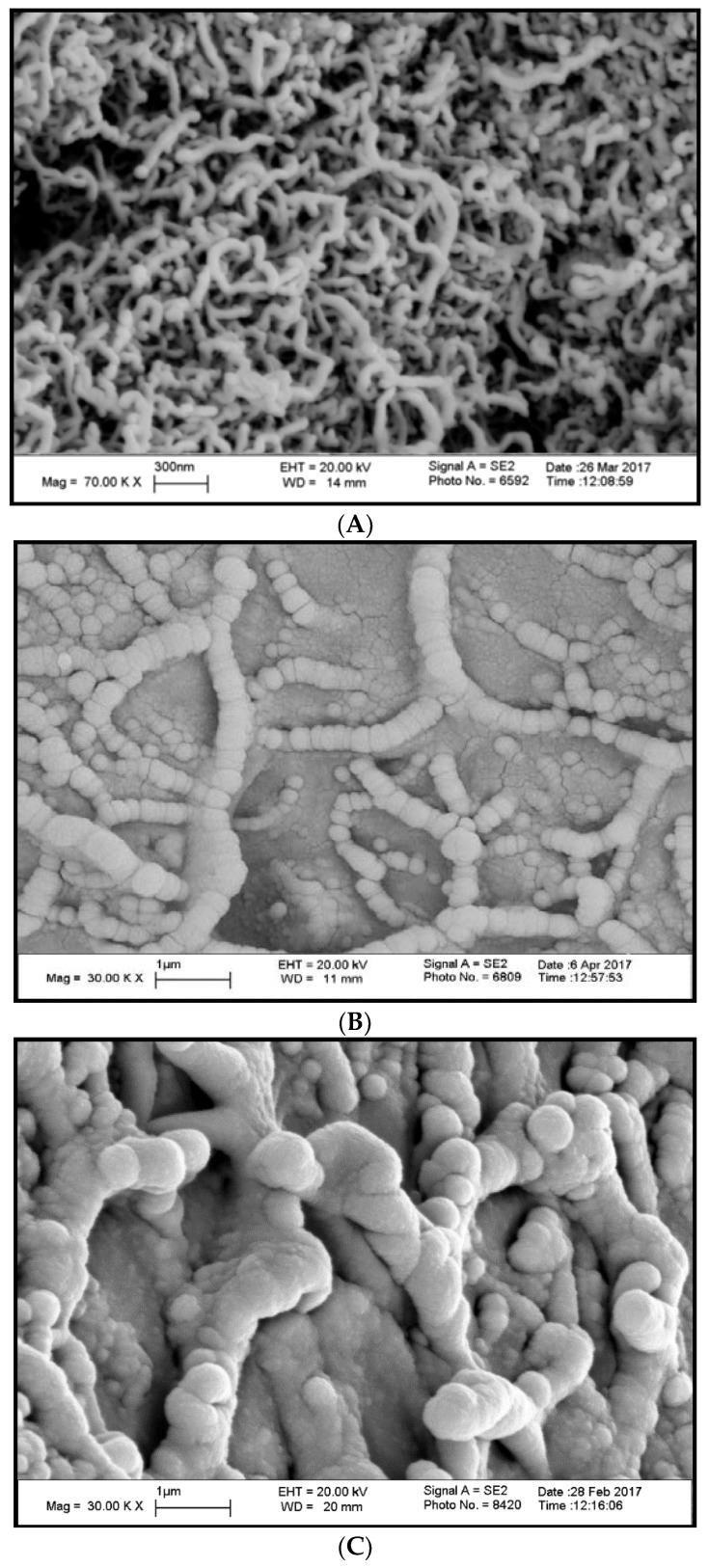
SEM micrographs: CNT pristine (**A**), Ar-CNT-P (**B**) and Ar-CNT-C (**C**) composites with 5 wt % of CNTs.

In order to access the state of the primary agglomerates before incorporating in the matrix, SEM was used to observe the CNTs in pure form. Figure 3 shows the SEM micrographs of the pristine CNTs together with the two types of aramid hybrids with 5.0 Wt. % loadings of CNTs.

The pristine multiwalled CNTs are seen in the form of small aggregates and tangled clusters. The outer diameter of these CNTs varied from 10–20 nm. The size of agglomerates was reduced significantly after sonification. As is clear from Figure 3, most of the CNTs are well dispersed in the polymeric matrix with only a few restacks. The aramid chains on the CNT surface are further linked up/entangled with the other chains in the matrix. However, there was a major change in the morphology and structure of Ar-CNT-C composites in comparison to Ar-CNT-P where pristine CNTs were used. In chemically-bonded hybrids, the surface of CNTs is imbibed heavily with the polymer chains and the thicknesses of the tubes have increased tremendously due to the complete covering by polymer chains. The CNTs are isolated, well dispersed and exist in the form of a thicker network due to cross-linking/wrapping of polymer chains on their surface. The outer diameter of the original CNTs was around 10–20 nm, as received from the supplier, but as a consequence of imbibition and coverage their outer diameter in the Ar-CNT-C composites has increased to 350–500 nm in the case where the functionalized CNTs were used. The diameter of the CNTs is on the same size scale as the radii of the aramid chains and supports the formation of large helices around the tubes. Such a core–shell structure of CNT covered and bonded extensively with the polymer chains can help greatly in the transfer of stress from matrix to the reinforcement, thus improving the mechanical properties in the composite material. The increase in external diameter in the case of AR-CNT-P is lower, as seen in Figure 3, as it involved only physical bonding between the conjugated structure of CNTs and aramid chains.

### 3.4. Atomic Force Microscopy

**Figure 4:**
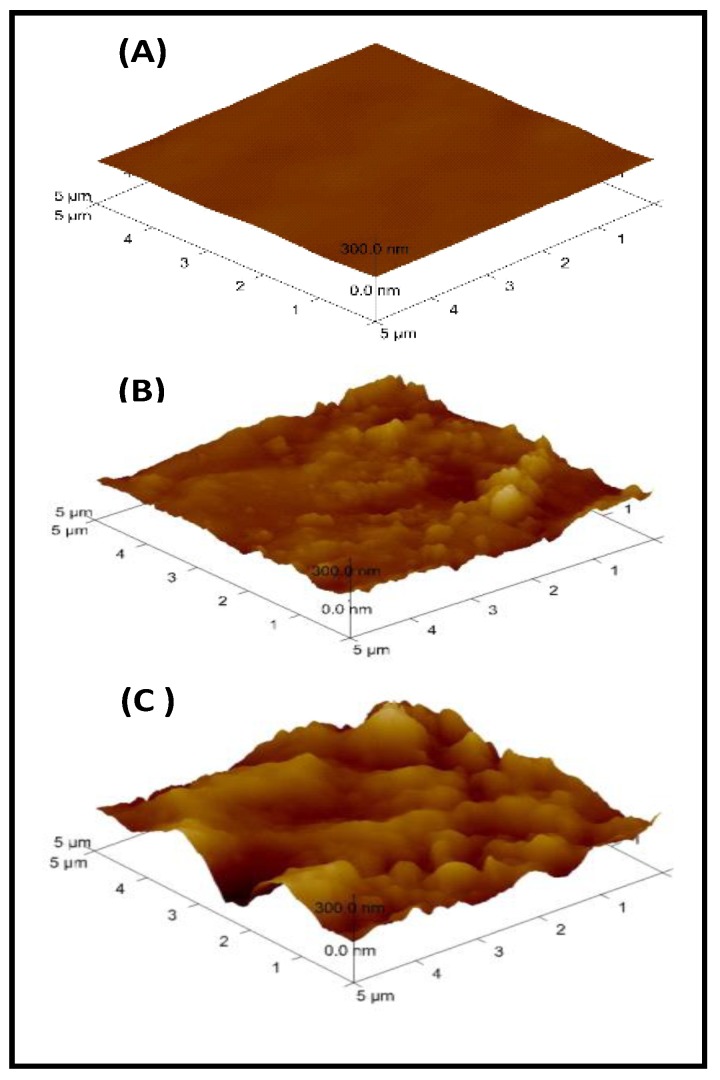
Atomic force microscopy (AFM) Images: CNT pristine (**A**), Ar-CNT-P (**B**) and Ar-CNT-C (**C**) composites with 7.5 wt % of CNTs.

Figure 4 shows the topology of the surface of pure aramid and the composite films with 7.5 wt % loading of CNTs. The surface of the pure film is seen as smooth and flat, but the roughness of the surface has increased considerably on incorporating CNTs in the resin, which is consistent with the SEM observations. The distribution of higher points varies with clusters varying in height. The vertical scale was kept at 300 nm level. The surface morphology of the Ar-CNT-C composite film is different from that of Ar-CNT-P films. In Ar-CNT-P film, the CNT network is in condensed form with some sharp peaks, whereas it is in the form of more dispersed clouds rather than the sharp peaks in bonded hybrids. In Ar-CNT-C hybrid film the CNT domain entering the organic network gradually or vice versa; thus, two phases became intermingled with each other. The blurred or diffused surface of the film allows us to believe that, in Ar-CNT-C composites, the domains became interpenetrated or linked through a diffused interface with the organic network. The effect of changing morphology and commingling of the phases can influence the mechanical behavior of the material. The achievement of such morphology due to better interfacial adhesion and the formation of hybrid morphologies, the stress-transfer between the phases under the condition of stress is improved, thus giving better mechanical strength.

### 3.5. Dynamic Mechanical Analysis (DMA)

**Figure 5:**
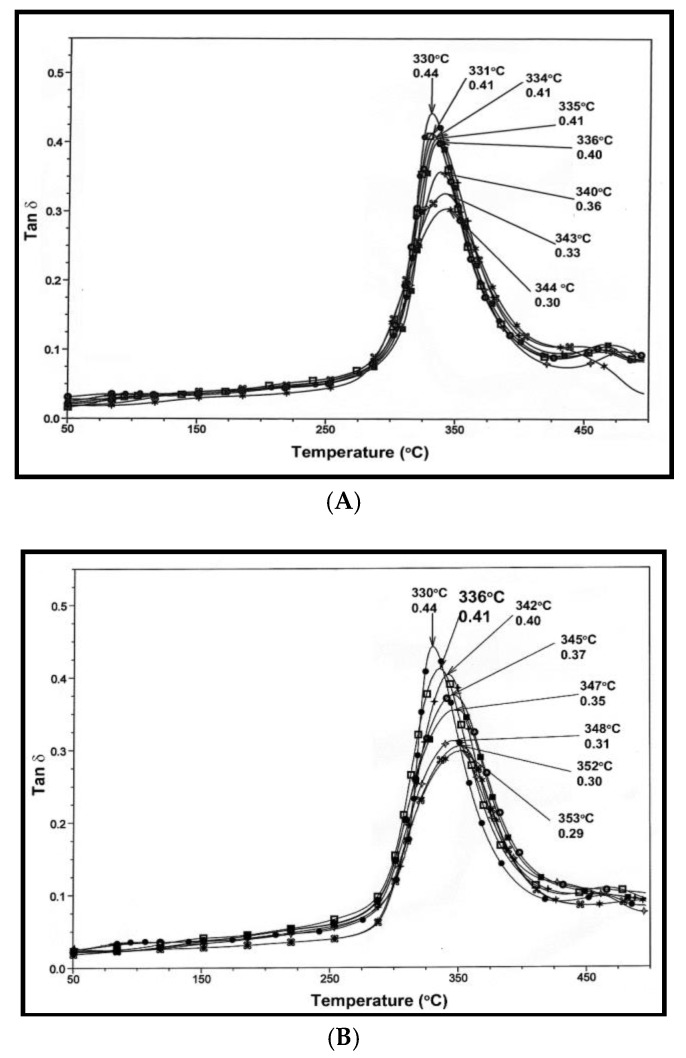
Temperature variation of tan δ: Ar-CNT-P (**A**) and AR-CNT-C (**B**) composites; CNT wt %: 0 (●), 0.5 (□), 1 (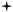), 1.5 (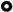), 2 (■), 2.5 (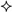), 5 (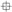), 7.5 (*).

**Figure 6:**
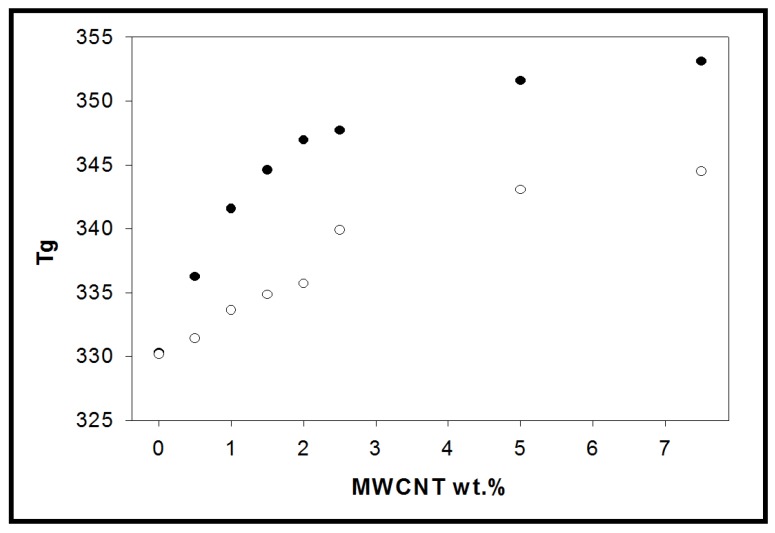
Glass transition temperature (T_g_) variation with CNT loading in Ar-CNT-P (◯) and Ar-CNT-C (●) composites.

Thermal–mechanical properties of Ar-CNT films were studied by DMA. Figure 5 shows the temperature variation of tan δ for the both types of composites. The T_g_ values associated with α-relaxations were measured from the maxima of these curves. These values were shifted in both cases to a higher temperature region. The physical interactions of the CNTs with the matrix in the case of Ar-CNT-P hybrids are due to the aromatic rings contained in the aramid chains that form physical bonding with the surface of CNTs through π-π stacking, but the shift is more in the case of Ar-CNT-C composites where the CNTs were chemically bonded with the polymer matrix (Figure 6).

The strengthening of the interface takes place along the CNTs due to immobilized chains, and, with increased loading of CNTs, the fraction of the volume at interface increases and a higher fraction of the polymer chains is immobilized. The T_g_ for the neat aramid film is around 331 °C and the maximum value of T_g_ obtained in these hybrids with 7.5 wt % addition of pristine CNTs is 344 °C. More increase in T_g_ with the maximum value of 351 °C in the case of a bonded system suggests the rotational mobility of the chains has been restricted due to chemical linkage of these on the CNT surface. This is also clear from the height of tan δ curves where, in fact, relatively more dampening is observed (Figure 5) on increasing the CNT content in the case of Ar-CNT-C hybrids as compared to the Ar-CNT-P.

The cited literature on similar composites reported over the last ten years, however, shows many contradictions. Rathore et al. [32] studied the properties of CNT-embedded epoxy composite and found the T_g_ to decrease on addition of both the pristine and carboxyl functionalized CNTs. Similar results were reported [33] for the epoxy composites by Masoud et al., where the thermal decomposition temperature of epoxies was found to increase but the values of T_g_ decreased on addition of CNTs. Hameed et al. [34] and Zhou et al. both separately studied the thermal–mechanical properties of epoxy-CNT composite using functionalized CNT and found a slight increase in the T_g_ value. Using dynamic mechanical analysis on polycarbonate-CNTs composites prepared by different methods [36,37] using both functionalized and non-functionalized, a depression in the T_g_ has been reported, and the decrease was found to be even more in the case of functionalized CNTs. The in-situ polymerization of methyl methacrylate [38] in the presence of varying amounts of CNTs have shown an increase in the T_g_.

Different techniques have been used in the past for the preparation of these composites, and we find that calendaring or shear mixing techniques are generally not very helpful in improving the thermal mechanical properties. Surface modification of CNTs creating physical interaction with the matrix help to improve the viscoelastic behavior [19,20]. The in-situ polymerization of the monomer in the presence of functionalized CNTs, however, gives much better results, but precautions have to be observed even in this case as the non-stoichiometric concentrations of the monomer and the functionalized CNTs can cause reductions in the T_g_. The selective sorption of one component and a surplus of the other acts as plasticizer on the CNT surface which can cause a decrease in the relaxation behavior of the filled system as observed in many reported polymer-CNT hybrids systems of the past.

Although direct measurements of interphase region are not available, the quantitative evidence in our work can be seen from the SEM images shown in Figure 3. The large extent of the interphase gradient on CNT loadings has penetrated in the matrix to different extents depending on the physical or chemical interactions, thus forming a percolating altered polymer network, and the extent of structural gradient in a polymer matrix is responsible for an increase in the T_g_ of the aramids to different extents as shown in Figure 6.

**Figure 7:**
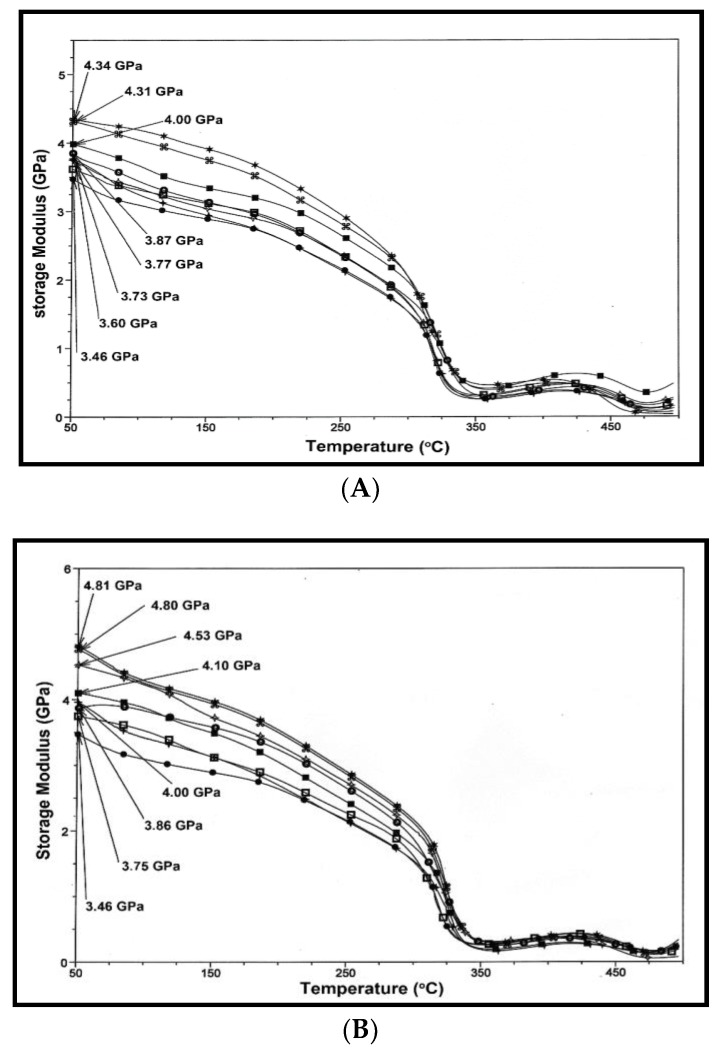
Temperature variation of Storage Modulus: Ar-CNT-P (**A**) and AR-CNT-C (**B**) composites; CNT wt %: 0 (●), 0.5 (□), 1 (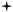), 1.5 (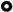), 2 (■), 2.5 (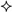), 5 (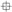) and 7.5 (*).

**Figure 8:**
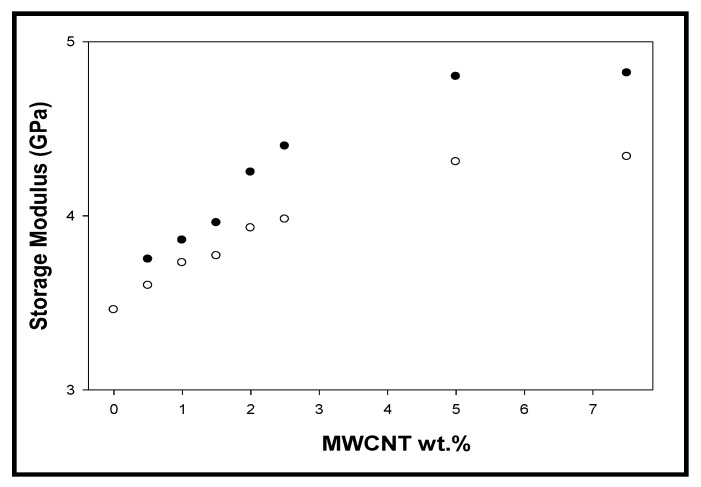
Storage modulus variation with CNT loading in Ar-CNT-P (◯) and Ar-CNT-C (●) composites.

The variation of the storage modulus with temperature on CNT loading for both hybrid systems is given in Figure 7. The modulus of neat aramid at 50 °C (in the glassy region) is around 3.46 GPa, and with CNTs loading it increases in both systems. The maximum value for the Ar-CNT-C system was 4.81 GPa in comparison to the 4.34 GPa for the AR-CNT-P system with 7.5 wt % loading of CNTs. The secondary or primary bond interactions between the CNT and the aramid matrix provide the load-transfer mechanism which is responsible for improvement in the storage modulus values. As the temperature is increased, i.e., it shifts to the rubbery region, the modulus values decrease in both systems by almost an order of magnitude, but these still remain higher than the neat matrix even in this region. A further increase in the modulus around 400 °C seems due to the cyclic stress which is induced during measurements taken in the tensile mode, which may cause some alignment in the polymer chains. However, as the polymer softens above 480 °C and a sharp drop in the E’ values is witnessed, in general, we found a higher increase in modulus for the chemically bonded hybrids in comparison to the physically associated system (Figure 8).

### 3.6. Tensile Analysis

**Figure 9:**
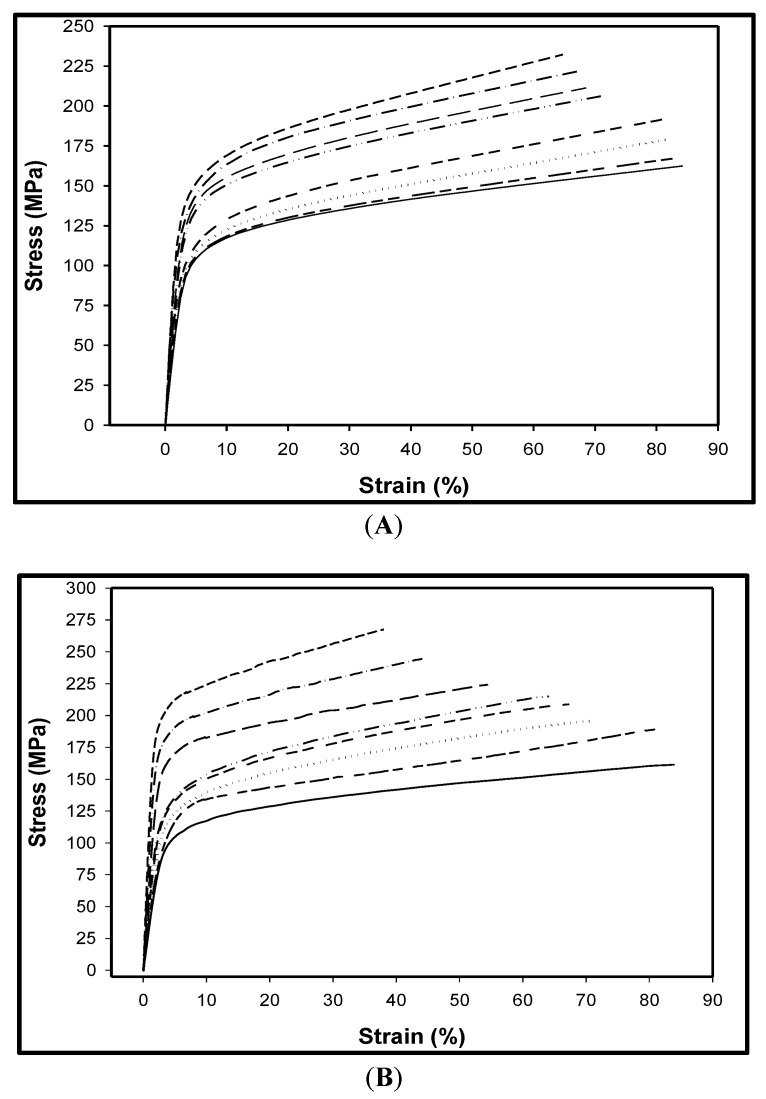
Stress vs strain curves of Ar-CNT-P (**A**) and AR-CNT-C (**B**) composite: CNT wt %: 0(**──**), 0.5 (− −), 1(····), 1.5 (− − − −), 2 (−··−), 2.5 (– – ), 5 (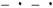), 7.5 (− − −).

**Figure 10:**
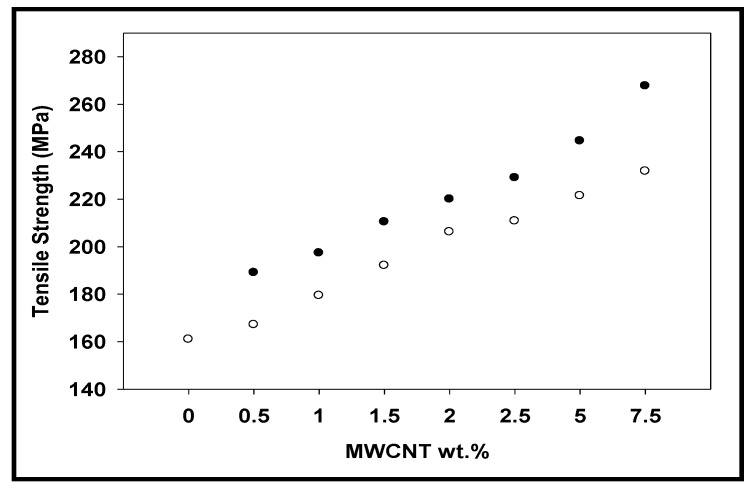
Tensile strength variation with CNT loading in Ar-CNT-P (◯) and Ar-CNT-C (●) composites.

**Figure 11:**
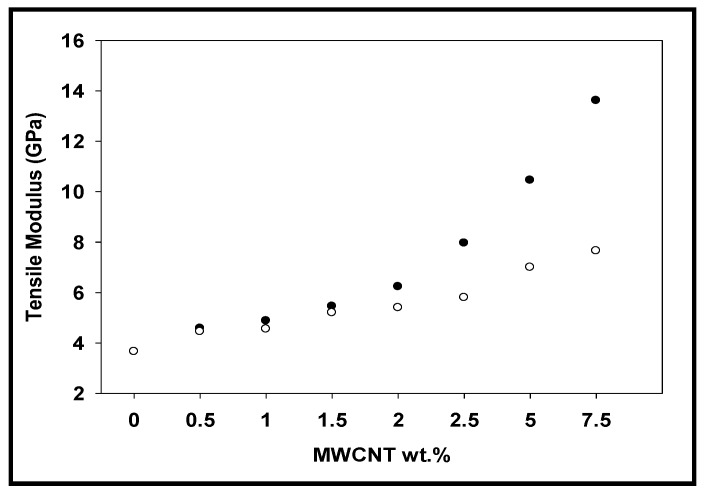
Tensile modulus variation with CNT loading in Ar-CNT-P (◯) and Ar-CNT-C (●) composites.

**Figure 12:**
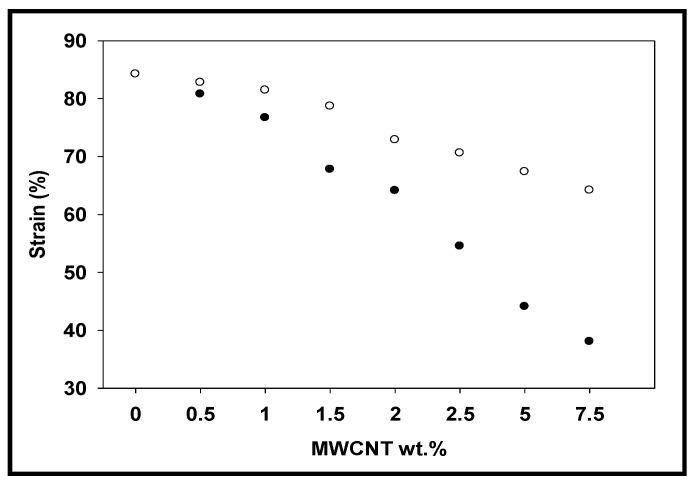
Strain variation with CNT loading in Ar-CNT-P (◯) and Ar-CNT-C (●) composites.

Tensile testing was performed to investigate the mechanical behavior of the Ar-CNT composite films. The stress–strain profiles for both types of hybrids are shown in Figure 9. The mechanical properties of composites films calculated from the instrument software are given in the Table 1. The tensile strength calculated from the maximum stress at the break point showed a higher increased in the case of A-CNT-C composites with the maximum value of 267.67 MPa (Figure 10). The modulus value for the neat matrix was 3.65 GPa which also showed a significant increase on chemical binding with CNTs, and the maximum value recorded with 7.5 wt % loading was 13.61 GPa. The increase in the case of pristine CNT was less than the value recorded for similar loading, which was 7.65 GPa (Figure 11) The elongation at break, however, decreased with the introduction of CNTs, and this decrease was found even more in case of Ar-CNT-C composites indicating these composites to be more rigid as compared to Ar-CNT-P system (Figure 12).

The nature of the interphase region of polymer chains developed in the vicinity of the CNTs has influenced greatly the properties of the polymer. In conventional fiber or particulate composites, the interphase is mainly considered to contribute to the load transfer effect; however, due to the large surface to volume ratio of CNTs, the magnitude of interphase generated in the matrix was substantial as already seen from the increase in the outer diameter of the CNTs in the scattering electron micrographs (Figure 3).

The chemical binding of the matrix chains on the surface of MWCNTs resulted in good interfacial adhesion between two phases, as well as the better dispersion of the CNTs in the aramid matrix. This is due to strong chemical bonding between the phases, whereas in the physically bonded Ar-CNT-P system, the slippage of the polymer chain at the interface was more probable under the tensile stress.

In the past, melt mixing/blending has been exploited for the dispersion of CNTs in various thermoplastic matrices [13,14,15,16,17], but these methods are not as successful, as the decomposition of the polymer chain occurs during the processing and the dispersion problems are not solved due to increased viscosity of the resin. Solution blending with high molecular weight polymers has also been tried, but the pristine CNT does not give significant improvement. In this work, we have used an in-situ polymerization technique where the CNTs and the monomers were mixed and then the reaction between the two phases as well as the polymerization proceeded together. This has ensured better dispersion as well as the binding of CNTs with the matrix chains, and we were able to use much higher loading without any phase separation.

A careful choice of using aromatic polyamides as a matrix in the present work instead of an aliphatic polyamide chains used [30,56,57] in the recent past has given much better results. The reason for this is the presence of phenyl groups in the aramid chains. The phenyl groups can form physical bonding due to van der Waals interactions with the surface of CNTs through π-π stacking. We were, therefore, able to compatibilize the system even using the pristine CNTs in the Ar-CNT-P composites, resulting a substantial increase in the mechanical strength. The functional groups of CNTs and their chemical reaction help a great deal in the interfacial load transfer via CNT/polymer chain bonding, thus leading to much higher improvement in mechanical properties, and such promising lightweight materials with a tensile strength equal to 267 MPa can be considered for various structural applications in the high tech. industry.

### 3.7. Electrical Properties

The variation of electrical conductivity of these composite films with a closer gradient in CNT loadings was studied, and an abrupt change in the electrical conductivity for the Ar-CNT-P composites was observed (Table 2) at 3.5 wt % with a value 5.12 × 10^−5^ S cm^−1^, suggesting a percolation threshold in the electrical conductivity in these films. With an increase in the CNT concentration, this value increased to 9.78 × 10^−5^ S cm^−1^ for the 5.0 wt %. The maximum value of 2.78 × 10^−4^ S cm^−1^ was obtained with 7.5 wt % of CNTs. The electrical conductivity in such composites depends on various factors: i.e., the type and amount of electrically conductive fillers, nature of polymer used, dispersion of CNT in the matrix and its ability to conglomerate. The percolation threshold of CNT/Nylon-6 composite has been reported at 4–6 wt % of CNTs [58]. The crystallization-induced phase separation of CNTs in Nylon-6 seems to be the reason for low conductivity, whereas the kinks produced in the aramid chain by the meta-linkage in the para type of aramid chain in the present work helped in increasing the amorphous nature of the matrix and thus decreasing the percolation threshold slightly. The electrical behavior of Ar-CNT-C composites could not be measured using the four-point measurement technique in the range of present CNT loadings. Functionalization of CNTs seems to have disrupted the electrical paths in these composites films. The impact of disruption in extended π conjugation combined with conversion of carbon hybridization from SP^2^ to SP^3^ had no effect on the mechanical properties; the effect on electrical properties, however, seems to be profound.

Using carbon black or carbon fiber-based composites [59,60], the electrical percolation threshold values are quite high: i.e., the filler content required is nearly 10–20 wt %, whereas in the Ar-CN-P composites with better thermal–mechanical properties, the electrical percolation threshold can be achieved at very low loadings, and these, therefore, can substitute as fillers for the materials required for EMI shielding and as antistatic materials.

### 3.8. Water Absorption

**Figure 13:**
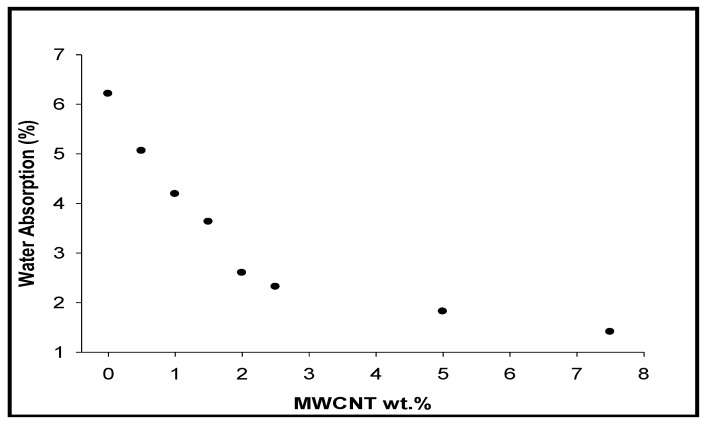
Water absorption (%) in the Ar-CNT-P composite films vs CNT loading.

Figure 13 shows water absorption as a function of CNT loading in the AR-CNT-P hybrids. The water absorption in the films has greatly been reduced. The problem with Kevlar or Nomax types of material is that these absorb water which can affect their mechanical strength. However, the low mass density of the CNTs together with their hydrophobic character greatly reduce this tendency in such films. The diffusion of water molecules becomes more difficult in such composite films due to the presence of the CNT network spread on the surface of the film. The increase in the weight of aramid neat film after 48 h at 25 C was 6.21%, whereas it was reduced to 1.41 with 7.5 wt % loading of pristine CNTs. The absorption rate, however, was found to be higher in the case of Ar-CNT-C hybrids, whereas it was 3.22% with similar loadings of 7.5 wt %. This seems due to the presence of few polar groups in the case of functionalized CNTs.

### 3.9. Thermogravimetric Analysis

**Figure 14:**
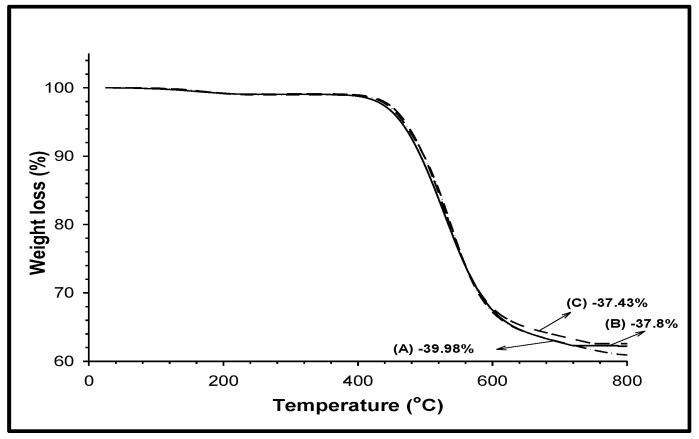
TGA thermograms; Ar pure (**A**), Ar-CNT-C (**B**) and Ar-CNT-P(**C**) composites with 5 wt % CNT.

**Figure 15:**
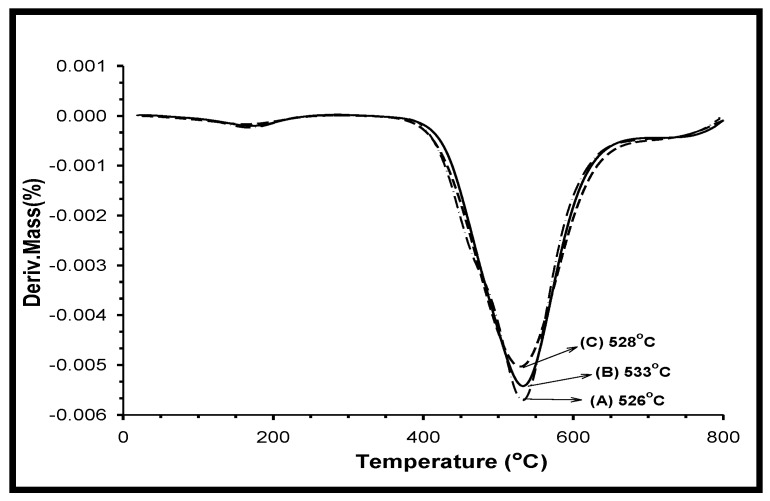
DTGA: Ar pure (**A**); Ar-CNT-C (**B**) and Ar-CNT-P (**C**) composites with 5 wt % CNT.

Figure 14 shows the TGA curves for the neat aramid matrix along with 5 wt % CNT loadings both for the physically and chemically-bonded hybrids. The temperature involving maximum degradation of pure aramid as determined from the DTGA curve (Figure 15) is around 526 °C. In the case of Ar-CNT-P composites, there was a small increase in T_d_ to 528 °C, whereas this temperature for the Ar-CNT-C composites was 533 °C. The possible mechanism for the improved thermal stability seems to be the thermal conductivity due to CNTs in the polymer matrix, which has facilitated heat dissipation from within the matrix. Dispersed CNTs may also hinder the flux of degradation products which may also delay the degradation process. Though the observed improvement in thermal stability is nominal as the armid chain itself is very stable, the CNTs can also be useful as an efficient fire-retardant when mixed in polymer matrix.

## 4. Conclusions

An in-situ polymerization technique was successfully used to chemically link the high molecular weight aramid chains derived from the Kevlar/Nomex copolymer with acid-functionalized MWCNTs. The following are the main conclusions derived from this work.

1. With a careful choice of matrix, having aromatic rings like the aramid chains, it is possible to achieve physical bonding with the conjugated surface on the pristine CNTs through π-π stacking.
2. The interfacial interactions can further be increased by developing a chemical linkage between the matrix and the CNTs using in situ polymerization of the monomers and the functionalized CNTs.
3. Chemically bonded composites result in higher mechanical properties in comparison to the physically associated composites using pristine CNTs.
4. The low electrical percolation threshold is observed at 3.5 wt % of CNTs in the case of the composites when the pristine CNTs were used, whereas the chemical functionalization of CNTs with the matrix seems to have disrupted the electrical paths.
5. Water absorption capacity of aramid matrix decreases with the loading of pristine CNTs due to their low mass density and their hydrophobic nature.
6. Depending on the nature of application, both types of light-weight polymeric nano-composites with a high glass transition temperature, tensile strength and thermal stability, can be useful for various applications including EMI shielding and antistatic materials for low-observable applications.

## Figures and Tables

**Table 1:** Tensile Properties of Ar-CNT-P and Ar-CNT-C Composites. MWCNT: multiwalled carbon nanotubes.

Sample	MWCNT wt %	Tensile Modulus (GPa)		Tensile Strength (MPa)		Strain at Break (%)	
-	-	Ar-CNT-P	Ar-CNT-C	Ar-CNT-P	Ar-CNT-C	Ar-CNT-P	Ar-CNT-C
Ar neat	0	3.65	3.65	161.10	161.00	83.85	83.85
Ar-CNT-0.5%	0.5	4.40	4.58	167.42	189.03	82.90	80.77
Ar-CNT-1%	1	4.50	4.87	179.42	197.32	81.61	73.20
Ar-CNT-1.5%	1.5	5.30	5.45	192.05	210.38	81.02	67.78
Ar-CNT-2%	2	5.36	6.23	206.19	216.18	70.40	64.11
Ar-CNT-2.5%	2.5	5.70	7.96	211.74	224.35	68.52	54.53
Ar-CNT-5%	5	7.37	10.45	221.42	244.50	67.10	44.08
Ar-CNT-7.5%	7.5	7.65	13.61	232.45	267.67	64.00	38.05

**Table 2:** Variation of electrical conductivity with CNT loading in Ar-CNT-P composites.

CNT wt % in the Aramid Matrix	Electrical Conductivity (S cm^−1^)
3.5	5.12 × 10^−5^
5.0	9.78 × 10^−5^
7.5	2.78 × 10^−4^
